# Microevolution and Adaptive Strategy of Psychrophilic Species *Flavobacterium bomense* sp. nov. Isolated From Glaciers

**DOI:** 10.3389/fmicb.2019.01069

**Published:** 2019-05-22

**Authors:** Qing Liu, Hong-Can Liu, Yu-Guang Zhou, Yu-Hua Xin

**Affiliations:** ^1^ China General Microbiological Culture Collection Center (CGMCC), Institute of Microbiology, Chinese Academy of Sciences, Beijing, China; ^2^ State Key Laboratory of Microbial Resources, Institute of Microbiology, Chinese Academy of Sciences, Beijing, China

**Keywords:** *Flavobacterium bomense*, glacier-inhabiting bacteria, microevolution, cold adaptation, psychrophilic

## Abstract

Numerous mountain glaciers located on the Tibetan Plateau are inhabited by abundant microorganisms. The microorganisms on the glacier surface are exposed to the cold, barren, and high-ultraviolet radiation environments. Although the microbial community composition on glaciers has been revealed by high-throughput sequencing, little is known about the microevolution and adaptive strategy of certain bacterial populations. In this study, we used a polyphasic approach to determine the taxonomic status of 11 psychrophilic *Flavobacterium* strains isolated from glaciers on the Tibetan Plateau and performed a comparative genomic analysis. The phylogenetic tree based on the concatenated single-copy gene sequences showed the 11 strains clustered together, forming a distinct and novel clade in the genus *Flavobacterium*. The average nucleotide identity (ANI) values among these strains were higher than 96%. However, the values much lower than 90% between them and related species indicated that they represent a novel species and the name *Flavobacterium bomense* sp. nov. is proposed. The core and accessory genomes of strains in this new *Flavobacterium* species showed diverse distinct patterns of gene content and metabolism pathway. In order to infer the driving evolutionary forces of the core genomes, homologous recombination was found to contribute twice as much to nucleotide substitutions as mutations. A series of genes encoding proteins with known or predicted roles in cold adaptation were found in their genomes, for example, cold-shock protein, proteorhodopsin, osmoprotection, and membrane-related proteins. A comparative analysis of the group with optimal growth temperature (OGT) ≤ 20°C and the group with OGT > 20°C of the 32 *Flavobacterium* type strains and 11 new strains revealed multiple amino acid substitutions, including the decrease of the proline and glutamine content and the increase of the methionine and isoleucine content in the group with OGT ≤ 20°C, which may contribute to increased protein flexibility at low temperatures. Thus, this study discovered a novel *Flavobacterium* species in glaciers, which has high intraspecific diversity and multiple adaptation mechanisms that enable them to cope and thrive in extreme habitats.

## Introduction

Glaciers harbor large number of microorganisms, including viruses, bacteria, archaea, and microeukaryotes, which have the ability to survive at low temperatures (Boetius et al., 2015). Low temperature is an important evolutionary force in the diversification of microorganisms. The collection of these cold-adapted microbes and analysis of their genetic characteristics is very important for us to understand how they can live in such cold environment and how the high species diversity is maintained. In recent years, numerous studies have been conducted to determine the microbial community structure on glaciers (Liu et al., 2009, 2015a,b, 2017; Franzetti et al., 2013). These studies revealed the most abundant bacterial genera on glaciers, including *Flavobacterium*, *Arthrobacter*, *Hymenobacter*, *Deinococcus*, *Cryobacterium*, *Polaromonas*, and *Sphingomonas*.

The genus *Flavobacterium* was proposed by Bergey et al. (1923) with *Flavobacterium aquatile* as the type species. It belongs to the phylum *Bacteroidetes* (formerly the *Cytophaga*-*Flavobacterium*-*Bacteroides* group), and its description was emended by Bernardet et al. (1996). Currently, it contains 150 species and is the largest genus within the family *Flavobacteriaceae*. Members of *Flavobacterium* produce carotenoids and flexirubin-type pigments, which make them yellowish or orange (Reichenbach et al., 1980; Bernardet and Bowman, 2015). *Flavobacterium* strains live in a variety of environments throughout temperate and polar regions, including terrenes, lakes, oceans, glaciers, plants, animals, and so on (Bernardet and Bowman, 2015). Some strains isolated from animals are pathogenic. *Flavobacterium* was found to be one of the most abundant genera in the Arctic and Antarctic sea ice (Boetius et al., 2015). In a survey of bacterial diversity on the surfaces of mountain glaciers in China, *Flavobacterium* was one of the three most abundant genera with more than 5% of average abundance (Liu et al., 2015b). Up to now, a total of seven psychrophilic/psychrotolerant *Flavobacterium* species with validly published names have been collected from glaciers, including *F*. *collinsense* (Zhang et al., 2016), *F*. *glaciei* (Zhang et al., 2006), *F*. *noncentrifugens* (Zhu et al., 2013), *F*. *xinjiangense* (Zhu et al., 2003), *F*. *omnivorum* (Zhu et al., 2003), *F*. *sinopsychrotolerans* (Xu et al., 2011), *F*. *tiangeerense* (Xin et al., 2009), *F*. *xueshanense* (Dong et al., 2012), and *F*. *urumqiense* (Dong et al., 2012).

With the development of modern “omics” technologies, some cold-adaptation strategies of microorganisms have been revealed. These specific physiological mechanisms are associated with cellular membrane fluidity (Casanueva et al., 2010), compatible solutes (Welsh, 2000), antifreeze protein (Chattopadhyay, 2006), ice-binding proteins (Raymond et al., 2007, 2008), anti-nucleating proteins (Kawahara, 2002), cold-shock proteins (Phadtare, 2004), cold acclimation protein (Phadtare, 2004), DEAD-box RNA helicase (Kuhn, 2012), cold-active enzymes (Feller and Gerday, 2003; Damico et al., 2006; Feller, 2010; Kasana and Gulati, 2011), energy generation and conservation (Amato and Christner, 2009), and genome plasticity (Casanueva et al., 2010).

Although the development of high-throughput sequencing techniques enables us to analyze bacterial diversity and community composition in cold environments, questions remain about the microevolution of certain psychrophilic/psychrotolerant groups. For instance, to survive for a long time at low temperature, which kinds of adaptations to low temperature have evolved in these lineages? How do these bacteria endure the harsh conditions and dwell in cold environments? During a survey of bacterial diversity on the surface of four glaciers located in Bome County, Tibetan Autonomous Region, P.R. China, we collected 11 psychrophilic *Flavobacterium* strains. Using a polyphasic approach, we determined the taxonomic status of these strains and identified a novel species, which we proposed to be named *Flavobacterium bomense* sp. nov. Furthermore, the intraspecies diversity, driving forces of microevolution, and cold-adaptation strategies of these glacier-inhabiting *Flavobacterium* strains were analyzed by comparative genomics.

## Materials and Methods

### Bacterial Strains and Culture Conditions

A total of 11 strains were isolated from melt water and ice samples from the surface of the Laigu, Zepu, Renlongba, and Gawalong glaciers in Bome County, Tibetan Autonomous Region, P.R. China (Table 1). All isolates were picked up as yellow, round colonies from PYG (peptone, yeast extract, and glucose) medium (Liu et al., 2015a) and 1/4 R2A (BD Difco, Becton, Dickinson and Company, Franklin Lakes, NJ, USA) agar plate. Six type strains of closely related species, namely *F*. *urumqiense* CGMCC 1.9230^T^, *F*. *sinopsychrotolerans* CGMCC 1.8704^T^, *F*. *tiangeerense* CGMCC 1.6847^T^, *F*. *xueshanense* CGMCC 1.9227^T^, *F*. *omnivorum* CGMCC 1.2747^T^, and *F*. *frigidarium* CGMCC 1.9172^T^, were used as reference strains for comparative analysis. All strains were routinely incubated in PYG medium at 15°C.

**Table 1:** Information of the *Flavobacterium* strains analyzed in this study and GenBank accession nos. of the 16S rRNA gene.

Strain	CGMCC no.	Isolation source	Isolation medium	Glacier	Location	Altitude (m)	GenBank accession no.
LB2P53	1.11357	Ice	PYG	Laigu	29.3087826 N, 96.8186951 E	3931.6	MK346152
LS1R10	1.11580	Melt water	1/4 R2A	Laigu	29.3087826 N, 96.8186951 E	3931.6	MK346153
LS1P28	1.11664	Melt water	PYG	Laigu	29.3087826 N, 96.8186951 E	3931.6	MK346154
**RB1N8**^**T**^	**1.23902**	**Ice**	PYG	**Renlongba**	**29.2615929 N, 96.9359436 E**	**4651.7**	**MK346155**
ZB4P23	1.24058	Ice	PYG	Zepu	30.276556 N, 95.2508392 E	3454.6	MK346156
RSP15	1.24446	Melt water	PYG	Renlongba	29.2615929 N, 96.9359436 E	4651.7	MK346157
RSP46	1.24469	Melt water	PYG	Renlongba	29.2615929 N, 96.9359436 E	4651.7	MK346158
RSP49	1.24471	Melt water	PYG	Renlongba	29.2615929 N, 96.9359436 E	4651.7	MK346159
GSP6	1.24637	Melt water	PYG	Gawalong	29.7659264 N, 95.71035 E	3842.3	MK346160
GSP27	1.24647	Melt water	PYG	Gawalong	29.7659264 N, 95.71035 E	3842.3	MK346161
GSN2	1.24670	Melt water	PYG	Gawalong	29.7659264 N, 95.71035 E	3842.3	MK346162

### DNA Extraction, Amplification, and Sequencing

Genomic DNA was extracted using the Genomic DNA Rapid Isolation Kit for Bacterial Cell (BioDev-Tech, Co., Beijing, China) following the manufacturer’s instructions. The 16S rRNA gene was amplified and sequenced using the universal primers 27F and 1492R (Lane, 1991). Sequencing was performed using a PRISM 3730XL DNA analyzer (Applied Biosystems, Foster City, CA, USA) at SinoGenoMax Co. (Beijing, China). Whole genome sequencing of the 11 strains was performed using the Illumina HiSeq 4,000 platform (Illumina, San Diego, CA, USA) according to the manufacturer’s protocols. The assemblies of short reads were performed using the SPAdes 3.11 program with default parameters (Bankevich et al., 2012). The quality of the genomes was assessed by CheckM (Parks et al., 2015) and QUAST v5 (Mikheenko et al., 2018).

### Phylogenetic Analysis Based on 16S rRNA Genes

For identification of the new strains, the 16S rRNA gene sequences were submitted to the EzBioCloud server (Yoon et al., 2017) to search for their closely phylogenetic neighbors. After multiple sequence alignment of the 16S rRNA gene sequences by ClustalW (Thompson et al., 1994), the neighbor-joining (NJ) and maximum-likelihood (ML) trees were built with 1,000 bootstrap replicates using the MEGA V. 5.2 software (Tamura et al., 2011). Kimura’s two-parameter model (K2P) was used to calculate the genetic distances (Kimura, 1980). The GTR + G + I was selected as the best nucleotide substitution model for ML tree construction.

### Comparative Genomic Analysis

The draft genomes determined in this study were submitted for annotation using RASTkt (Brettin et al., 2015). To further confirm the taxonomic status of the new strains, a total of 32 whole genome sequences of related type strains were obtained from the NCBI genome database (Supplementary Table S1). For construction of species trees, single-copy orthologues were selected from genomic sequences using GET_HOMOLOGUES (Contreras-Moreira and Vinuesa, 2013) and GET_PHYLOMARKERS programs (Vinuesa et al., 2018). The alignments were generated by Clustal Omega software (Sievers and Higgins, 2014) implemented in GET_PHYLOMARKERS. Phylogenetic trees were generated by ML algorithms with a GTR + F + R5 model in the IQ-TREE software (Nguyen et al., 2015) based on the concatenated gene sequences with 1,000 bootstrap replicates. The average nucleotide identity (ANI) values were calculated by the GET_HOMOLOGUES program. The pan-genome analysis was performed using the BPGA tool with default parameters to determine the core and accessory genes (Chaudhari et al., 2016). Recombination analysis was performed with ClonalFrameML (Didelot and Wilson, 2015). Gene gain and loss rates were determined by the Count program using a birth-and-death model (Csuros, 2010) with the homologous table, which was inferred by using OMCL and bidirectional best hit (BDBH) methods implemented in GET_HOMOLOGUES package with the following parameters: 60% identity and 75% coverage.

### Phenotypic Characterization of Novel Species

A polyphasic taxonomic analysis of the 11 strains was performed in this study. The morphology of the colonies was determined after culturing the 11 strains on PYG agar for 7 days. The cellular morphology of strain RB1N8^T^ was examined by transmission electron microscopy using a JEM-1400 transmission electron microscope (JEOL Ltd., Tokyo, Japan). The growth at different temperatures (4, 15, 20, 22, 25, and 28°C), the tolerance to NaCl (0–4.0% (w/v) at 0.5% intervals), and pH values (ranging from pH 5.0 to 10.0 with 1 pH unit intervals) were tested in PYG broth. Hydrolyses of casein, starch, and Tween 80 were performed according to Smibert and Krieg (1994). The presence of flexirubin-type pigment was examined using 20% KOH (w/v). The utilization of sole carbon source, enzyme activities, and other phenotypic characteristics were tested using the API 20E, 20NE, ID 32 GN, and ZYM strips (bioMérieux, Marcy-l’Étoile, France) according to the manufacturer’s instructions.

For analysis of cellular fatty acid composition, the cells of the tested strains were harvested from colonies on the same sectors of the PYG plates after incubation at 15°C. The extraction of saponified and methylated fatty acids was performed according to the protocol of MIDI 6.0 system (Sasser, 1990). The samples were separated and identified on an Agilent 6,890 N gas chromatography system (Agilent Technologies, Santa Clara, CA, USA) using the TSBA6 database. Respiratory quinones and polar lipids were determined in cells of strain RB1N8^T^, which were harvested from PYG broth after incubation at 15°C for 5 days. The extracts of respiratory quinones and polar lipids were analyzed according to reported methods (Tindall et al., 2007).

### Nucleotide Sequence Accession Numbers

The GenBank accession numbers for the 16S rRNA gene sequences of the 11 *Flavobacterium* strains are MK346152–MK346162 (Table 1). The Whole Genome Shotgun projects have been deposited at DDBJ/ENA/GenBank under the accession numbers: RYDG00000000, RYDH00000000, RYDI00000000, RYDJ00000000, YDK00000000, RYDL00000000, RYDM00000000, RYDN00000000, RYDO00000000, RYDP00000000, and RYDF00000000, respectively (Supplementary Table S2).

## Results and Discussion

### Phylogenetic Analysis Based on 16S rRNA Gene Sequences

The average evolutionary divergence (K2P) over all the 11 16S rRNA gene sequence pairs was 0.001%. Accordingly, the sequence of strain RB1N8^T^ was selected as a representative to compare with the EzBioCloud database. The highest 16S rRNA gene sequence similarities were found between strain RB1N8^T^ and *F*. *xueshanense* Sr22^T^ (97.77%), *F*. *psychrolimnae* LMG 22018^T^ (97.49%), *F*. *fryxellicola* DSM 16209^T^ (97.49%), and *F*. *tiangeerense* 0563^T^ (97.40%), is lower than the 98.65% threshold value for species delineation (Kim et al., 2014), supporting the notion that the new group represents a novel species of the genus *Flavobacterium*. Phylogenetic analysis revealed that the 11 strains formed an independent lineage with strong bootstrap support of 100% (Supplementary Figure S1). Although some strains shared 100% sequence identities, strain diversity was noticeable in this new clade. The tree topologies constructed with ML algorithms were similar to that of the NJ trees (Supplementary Figure S2).

### Taxonomic Features and Phylogenetic Analysis Based on Genomes

Except for the genome sequence of strain GSN2, in which reads contamination was detected, the genome sequence assemblies of the other 10 strains are high quality (Supplementary Table S2). Thus, the genome sequence of strain GSN2 was ignored in the subsequent analysis, except for the single-copy gene phylogenetic reconstruction. These genomes were similar in size, ranging from 3.20 to 3.82 Mb. The DNA GC content was calculated to be from 34.91 to 35.04%, which is in agreement with the GC content of their closely related strains *F*. *xueshanense* Sr22^T^ (34.1%), *F*. *psychrolimnae* LMG 22018^T^ (34.2%), *F*. *fryxellicola* DSM 16209^T^ (34.6%), and *F*. *tiangeerense* 0563^T^ (33.6%). The ANI values among the 11 strains ranged from 96.89 to 99.17%, which are higher than the proposed cut-off value for species boundary (95–96%; Richter and Rosselló-Móra, 2009), indicating that they belong to one species. Furthermore, the ANI values between these strains and their closely related species with validly published names are much lower than 90% (Supplementary Table S1), revealing that this new *Flavobacterium* group represents a novel species.

**Figure 1:**
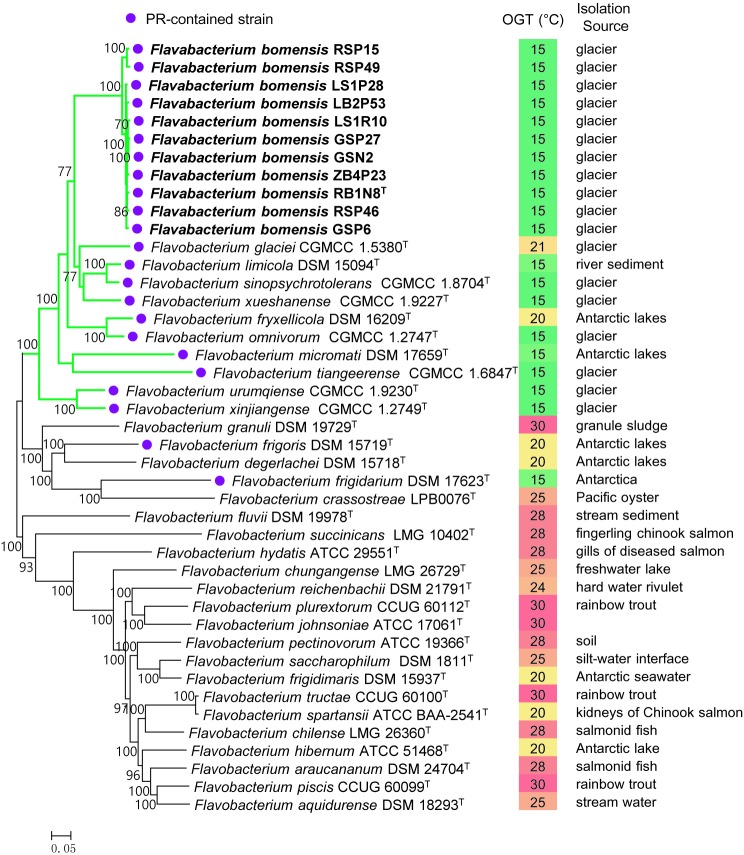
Phylogeny of the new strains and related species constructed from an ML analysis of the 230 concatenated single-copy gene sequences. Bootstrap values ≥70% based on 1,000 replicates are indicated at branch points. Bar = 0.05 nt substitutions per site. PR, proteorhodopsin; OGT, optimal growth temperature.

A total of 230 single-copy core genes were extracted from the genome sequences of 32 *Flavobacterium* type strains and 11 new strains. After sequence alignment, the concatenated single-copy gene sequences were used to construct ML and NJ phylogenetic trees with 1,000 bootstrap replicates (Figure 1), and the trees reconstructed by different methods showed identical topologies. The species tree clearly showed that the 11 tested strains clustered together, forming an independent and novel clade in the genus *Flavobacterium*. This novel clade and 10 other type strains, almost all of which were isolated from glaciers or Antarctic lakes, formed a larger lineage, revealing the diversification of *Flavobacterium* inhabiting in cold environments.

### Pan-Genome of the Novel Species

To gain a deeper understanding of the intraspecies genomic diversity of glacier-inhabiting *Flavobacterium* species, pan-genome analysis was performed. In this study, the pan-genome refers to the total number of orthologous gene families in the 10 strains isolated here with uncontaminated genome assemblies. The core pan-plot showed that the size of the pan-genome increased unlimitedly with the addition of new genomes (Supplementary Figure S3). The more genomes added, the more orthologous clusters produced. Thus, the pan-genome of the novel species was open. The core genome decreased as the genomes were added one by one and ultimately became relatively constant. A total of 2,269 genes formed the core gene pool of the novel species. Additionally, the total number of accessory genes comprised more than half of the pan-genome. Every strain contained a certain number of strain-specific genes (unique), and the number varied considerably (61–403) depending on individual strains, indicating that the ongoing genetic flow led to the generation of strain specificity (Supplementary Table S3).

**Figure 2:**
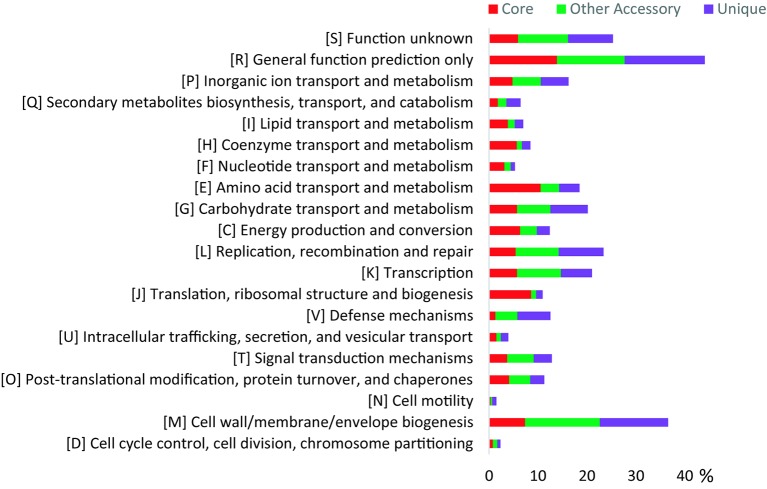
COG distribution for the pan-genome of 10 glacier-inhabiting *Flavobacterium* strains determined using the BPGA tool with default parameters. The *x* axis represents the relative percentage of the number of genes in each functional category.

Both the core genes and accessory genes were involved in all the categories of COG functions (Figure 2). However, most of the core genes was classified into the basic functions, such as “amino acid transport and metabolism [E],” “translation, ribosomal structure, and biogenesis [J],” “energy production and conversion [C],” “coenzyme transport and metabolism [H],” “lipid transport and metabolism [I],” and “nucleotide transport and metabolism [F].” Most of the accessory genes was related to functions of “cell wall/membrane/envelope biogenesis [M],” “transcription [K],” “carbohydrate transport and metabolism [G],” “replication, recombination, and repair [L],” and “signal transduction mechanisms [T],” which may contribute to adaptation to changing environments. In addition, the Kyoto Encyclopedia of Genes and Genomes (KEGG) analysis (Supplementary Figure S4) revealed that the unique genes were mainly involved in carbohydrate, amino acid, lipid, energy metabolism pathways, and signal transduction, especially carbohydrate metabolism, which corresponded with the variable ability of carbon source utilization tested by API ID 32 GN (Supplementary Table S4). These genomic characteristics indicated the diversity of metabolic pathways in different *Flavobacterium* strains. Thus, the pan-genome analysis showed the distinct patterns of gene content and metabolism pathway within this new *Flavobacterium* species.

### Microevolution of the Novel Species

**Figure 3:**
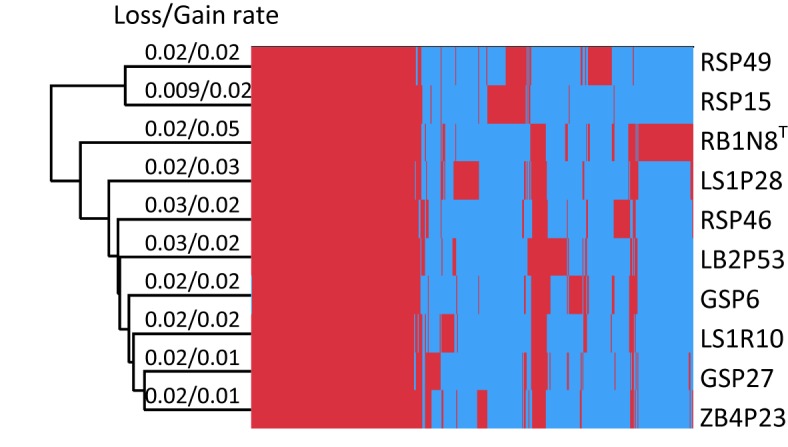
Hierarchical clustering of 10 strains based on the heatmap of the orthologous genes. The presence and absence of the orthologous genes for each strain are indicated in red and blue color, respectively.

In their evolutionary history, bacteria experience frequent gene gain and loss (Polz et al., 2013; Vos et al., 2015) or undergo homologous recombination and mutation within gene families (Didelot and Maiden, 2010), all of which may result in the variation of the pan-genome. These varied parts of the pan-genome are essential to the ability of the bacteria to survive in their individual habitat (McInerney et al., 2017). In order to investigate the evolutionary history of *Flavobacterium* strains, gene gain and loss rates were estimated (Figure 3). The results showed that the rates of gene gain and loss were about the same in every strain, except for strain RB1N8^T^, which gained more genes than it lost. The gene gain and loss of these *Flavobacterium* strains may have resulted from horizontal gene transfer (HGT). Bacteria could benefit from HGT, which would introduce new functions to adapt to the changing environment (Zhaxybayeva and Doolittle, 2011).

The contribution of homologous recombination and mutation to microevolution of the tested strains was further investigated by ClonalFrameML using a single-copy gene subset. The ratio of recombination to mutation rate (*ρ/θ*) and the ratio of nucleotide substitutions due to homologous recombination or mutation (*r/m*) were calculated based on 33 type strains and 10 new strains. Within species, *ρ/θ* and *r/m* values were 0.5064 and 2.1862, respectively. The values of *ρ/θ* and *r/m* between *Flavobacterium* species were 0.0052 and 0.3358. Accordingly, the rate of intraspecies recombination was much higher than that of interspecies. Although the *r/m* value was much lower than the values within many bacterial species (Vos and Didelot, 2009), homologous recombination still introduced more than twice the number of substitutions than that of mutations within the glacier-inhabiting *Flavobacterium* species.

Therefore, gain and loss of genetic material, recombination, and mutation all would result in the variations of the genomes. Such evolutionary events led to the formation of intraspecific diversity and phylogenetic cohesion of the novel *Flavobacterium* species.

### Insights of the Genome Sequences Linked to the Strategy of Living in Cold Environment

A number of known genes related to cold adaptation were identified in the novel species and compared with the genomes of two phylogenetic-related mesophiles, namely *F*. *granuli* DSM 19729^T^ and *F*. *crassostreae* LPB0076^T^ (Supplementary Table S5). The genes of proteorhodopsin, polysaccharide transporter, and ice-binding protein (IBPs) were absent in the two reference genomes, but the other adaptation-related genes were also found in the two mesophiles.

Proteorhodopsin (PR) is a light-dependent proton pump of bacteria, which can utilize solar irradiance to produce ATP (Béjà et al., 2001) and consequently can enhance the growth rate of some bacteria, but not all of them (Giovannoni et al., 2005; Gómez-Consarnau et al., 2007, 2016; Lami et al., 2009). Fuhrman et al. (2008) suggested that PR may contribute to the physiological adaptation of bacteria to stressful conditions. Comparative genome analysis revealed the presence of a gene encoding PR in all the new strains as well as in 12 closely related type strains, which were isolated from glaciers or the Antarctic. However, the PR gene was absent in the genomes of the 20 type strains of *Flavobacterium* analyzed in this study, most of which was mesophilic (Figure 1). Accordingly, the spread of the PR gene in cold environments informed our understanding of its ecological function. We inferred that harboring PR genes might be particularly advantageous to the survival of *Flavobacterium* bacteria in frigid and barren environments.

Extracellular polysaccharides (EPSs) serve as cryoprotectants in bacteria living in marine or other cold environments (Nichols et al., 2005; Reid et al., 2006). All the tested strains in this study contained one gene copy of *lptA*, *lptB*, *lptC*, *lptF*, and *lptG*, which are related to the transportation of polysaccharide out of the membrane, suggesting that these *Flavobacterium* strains could produce and export EPSs to protect the cells against the harm of low temperature.

Ice-binding proteins (IBPs) inhibit the growth of ice crystals inside and outside the cells. The production of IBPs could make the bacteria counteract deleterious effects under subzero conditions (Mangiagalli et al., 2017). One gene copy of IBP was observed in the 10 *Flavobacterium* strains. IBP genes have also been identified in other psychrophilic bacteria, such as Antarctic *Colwellia* sp. SLW05 (Raymond et al., 2007) and *Psychroflexus torquis* (Mangiagalli et al., 2017)

Sigma factors perform all initiation of transcription in bacteria. Except for the single essential housekeeping *σ* that promotes the transcription of thousands of genes, many *σ*s promote the transcription of specialized genes in response to a particular stress or stimulus (Feklistov et al., 2014). The *σ*^70^ factors are commonly related to gene transcription, stress response, cell development, and auxiliary metabolism. Multiple copies of the *σ*^70^ gene (*rpoD*) have been found to increase resistance to cold stress in psychrophilic bacteria (Riley et al., 2008; Mykytczuk et al., 2013; Dsouza et al., 2015). In this study, the gene *rpoD* was also found in the novel *Flavobacterium* strains. Additionally, in their genomes, there were 9–10 copies of the *rpoE* gene, whose product (*σ*^24^) was associated with regulating cellular responses to heat-shock and other stresses on cellular membrane and periplasmic proteins (Dsouza et al., 2015).

In cold environments, the solubility of O_2_ increases at low temperatures and more reactive oxygen species (ROS) are formed (Chattopadhyay, 2002). Thus, for survival under oxidative stress conditions, psychrophilic bacteria could remove the ROS through proteins encoded by some functional genes, such as *sodA* (superoxide dismutase), *katE* and *katG* (catalase), peroxiredoxin *bcp* types (thiol peroxidases), *osmC*/*ohr* (organic hydroperoxide reductase), *trxB* (thioredoxin reductase), and *trxA* (thiol-disulfide isomerase and thioredoxins). Several copies of these genes were found in the genomes of the novel glacier-inhabiting *Flavobacterium* strains, and the proteins encoded by these genes may contribute to their survival on surfaces of glaciers with low temperature and high UV radiation.

When bacteria are suddenly exposed to a cold environment, a number of physiological changes occur in the cells and a set of small molecule proteins is expressed. Genes encoding these cold-shock-inducible proteins, including cold-shock protein (*cspA*), ribosome-binding factor A (*rbfA*), translation initiation factors, IF-1 and IF-2 (*infA*, *infB*), polynucleotide phosphorylase (*pnp*), and transcription termination protein A (*nusA*), were identified in all 10 *Flavobacterium* strains. In these strains, just one gene copy of cold-shock protein (*cspA*) was identified. However, several copies were observed in Antarctic *Arthrobacter* (Dsouza et al., 2015), *Colwellia psychrerythraea* (Methé et al., 2005), *Psychrobacter arcticus* (Ayaladelrío et al., 2010), and *Shewanella oneidensis* (Gao et al., 2006). Carotenoid could stabilize the cellular membrane at low temperature and therefore contribute to the adaptation of carotenoid-pigmented bacteria to a cold environment (Fong et al., 2001; Dieser et al., 2010). The intact pathway of carotenoid biosynthesis was found in the 10 genomes, including the genes *idi* (isopentenyl-diphosphate delta-isomerase), *crtB* (phytoene synthase), *crtI* (phytoene dehydrogenase), *crtY* (lycopene beta cyclase), and *crtZ* (beta-carotene hydroxylase). The presence of these genes was consistent with the yellow color of the colonies of these strains.

Glycogen is thought to help bacteria resist to stressful condition of low temperature (Bresolin et al., 2006; Dalmasso et al., 2012). Three enzymes, glycogen synthase (*glgA*), glycogen branching enzyme (*glgB*), and glucose-1-phosphate adenylytransferase (*glgC*), participate in the biosynthesis of glycogen (Cifuente et al., 2016). In this study, one to two copies of these genes were found in the genomes of 10 tested strains, which would enable them to accumulate carbon and energy reserves to cope with the cold and barren environments.

Proline is an important organic metabolite and has been proposed to be a protective osmolyte (Hoffmann and Bremer, 2011). Genes involved in proline biosynthesis and Na+/proline symporter were found in the 10 tested genomes, but genes of other compatible solutes such as glycine betaine were absent.

Maintaining the permeability and fluidity of the cellular membrane is important for bacteria living in cold environments, and this could be achieved through the synthesis of polyunsaturated fatty acids (Methé et al., 2005). The genome analysis in this study revealed the presence of genes for fatty acid desaturases (des), which would introduce the unsaturated double bond into the saturated fatty acid. Concerning the cellular fatty acid compositions (%) of *Flavobacterium* strains, several polyunsaturated fatty acids, including iso-C_15:1_ G, C_15:1_ *ω*6*c*, iso-C_16:1_ H, C_17:1_ *ω*6*c*, C_18:1_ *ω*5*c*, summed in Feature 3 (C_16:1_ *ω*6*c* and/or C_16:1_ *ω*7*c*), summed in Feature 4 (iso-C_17:1_ I/ anteiso-C_17:1_ B), and summed in Feature 9 (iso-C_17:1_ *ω*9*c*/10-methyl C_16:0_), were detected (Supplementary Table S6).

In order to increase the tRNA flexibility, psychrophilic bacteria can posttranscriptionally incorporate dihydrouridine in the tRNA (Dalluge et al., 1997). Similar to some other cold-adapted bacterial groups (Saunders et al., 2003; Wang et al., 2008; Qin et al., 2014), two copies of the gene encoding tRNA-dihydrouridine synthase (*dusB*) were found in the *Flavobacterium* strains, which could increase the conformational flexibility of their tRNAs to guarantee their survival in cold glaciers.

The system of Clustered Regularly Interspaced Short Palindromic Repeats (CRISPR) and its associated proteins (Cas) serve as an adaptive immune system for protecting prokaryotes against viral predators and foreign invaders (Hille and Charpentier, 2016). CRISPR-associated proteins Cas1 and Cas2, CRISPR-associated endonuclease Cas9, and CRISPR repeats (35–36 bp) were found present in the genomes of these *Flavobacterium* strains, indicating that the CRISPR-Cas system of *Flavobacterium* strains belonged to Type II system. There were 9–45 CRISPR repeats depending on the particular strain, showing a variety of CRISPR in the *Flavobacterium* strains. Some researchers have isolated bacteriophages from polar regions, deep sea, permafrost regions, high latitude lakes, and glaciers (Sawstrom et al., 2007; Li et al., 2016). Thus, the CRISPR-Cas systems found in these genomes indicate that viruses are also an important part of the glacier habitat.

**Figure 4:**
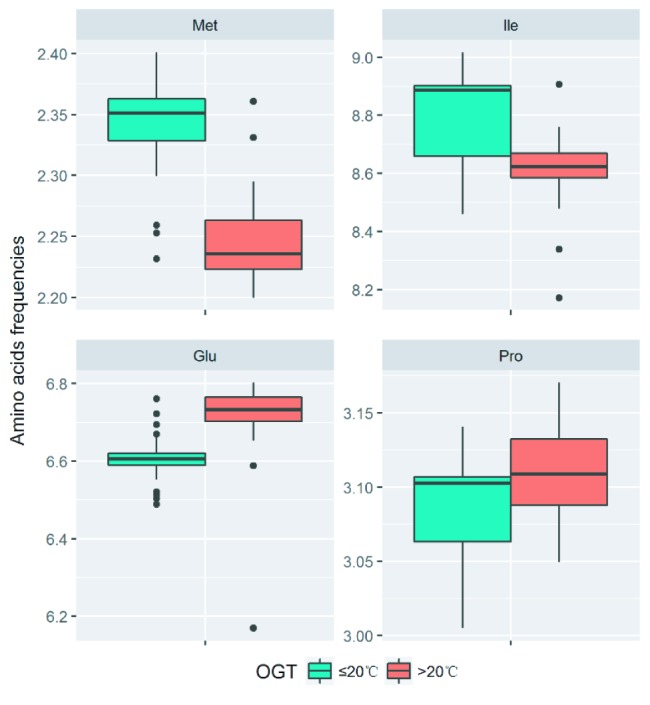
Difference of amino acid residue frequencies between the two groups of strains with OGT ≤ 20°C and OGT > 20°C in the genus *Flavobacterium*. The boxplot was produced in “ggplot2” package implemented in R. The significant differences were identified using Student’s *t* test.

At low temperatures, the most important property for proteins is the maintenance of sufficient flexibility, which can increase their interactions with substrates and reduce the required activation energy. The analysis of amino acid components revealed that the decrease in the number of proline residues (Fields, 2001), the introduction of methionine residues in the place of other buried hydrophobic residues (Marshall, 1997), and the decrease in the number of glutamine residues (Damico et al., 2006) were the strategies to increase the flexibility in cold-adapted proteins. In order to analyze the amino acid substitutions in proteins of strains with different optimal growth temperatures (OGT), the amino acid frequencies of the single-copy gene alignments of the 32 *Flavobacterium* type strains and 11 new strains were calculated. The OGT information of the type strains was acquired from the online catalogues of DSMZ^1^, JCM^2^, and CGMCC^3^. The strains with OGT ≤ 20°C contain more methionine, while the strains with OGT >20°C contain more proline and glutamine. Additionally, more isoleucine was found in the group with OGT ≤ 20°C. Our results suggested that the *Flavobacterium* strains may employed multiple amino acid substitutions to decrease protein stability and increase protein flexibility, which make the proteins more active at low temperatures (Figure 4).

### Phenotypic and Chemotaxonomic Characteristics

The new species of *Flavobacterium* was analyzed using phenotypic and chemotaxonomic methods. These strains were found to be Gram-negative, catalase and oxidase-positive, non-motile rods with yellow, and round colonies. No spores or flagella were detected (Supplementary Figure S5). These strains could grow in a temperature range of 0–22°C, and the maximum growth temperature for most of them was below 20°C (Supplementary Table S7). They were therefore considered to be a group of psychrophiles. They showed growth in a NaCl concentration range of 0–2.0/2.5 and a pH value range of 6.0/7.0–8.0/9.0, varying with different strains. Flexirubin-type pigment was absent. These strains could be easily distinguished from the type strains of their closely related species by their physiological and biochemical characteristics (Supplementary Table S7).

The profiles of cellular fatty acids in novel strains and reference strains are shown in Supplementary Table S6. The cellular fatty acid composition of novel strains and related type strains were similar, although there were some minor differences in certain components. The main fatty acids of the novel strains were summed Feature 3 (C_16:1_ *ω*6*c* and/or C_16:1_ *ω*7*c*, 17.4–33.8%) and iso-C_15:0_ (13.9–17.4%). The major hydroxyl fatty acids were iso-C_15:0_ 3-OH (5.9–8.3%), iso-C_16:0_ 3-OH (3.7–6.2%), and iso-C_17:0_ 3-OH (4.4–7.6%). Menaquinone 6 (MK-6) was the only isoprenoid quinone detected in strain RB1N8^T^, which is consistent with the other members of the genus *Flavobacterium* (Bernardet and Bowman, 2015). Polar lipids of RB1N8^T^ were phosphatidylethanolamine (PE), three unidentified polar lipids, and four unidentified aminolipids (Supplementary Figure S6), which is similar to the polar lipid profiles of other species of *Flavobacterium* (Bernardet and Bowman, 2015).

## Conclusion

In this study, 11 bacterial strains isolated from glaciers in China were classified as a novel *Flavobacterium* species using a polyphasic taxonomy method, for which the name *Flavobacterium bomensis* sp. nov. is proposed. A comparative genomic analysis of these strains was performed. Their pan-genome was open, and every genome contained some unique genes, which showed the diversity of gene content and metabolic pathways in this species. The driving forces of microevolution were also investigated. The results revealed that the accessory genomes of the new species gained and lost genes at certain rates, while homologous recombination showed a twofold higher contribution to nucleotide substitutions than mutation in their core genomes. In addition, all genomes harbored numerous genes related to adaptation to cold and harsh environments. These genes were involved in various adaptive aspects, such as carotenoid biosynthesis, carbon and energy reserves, the fluidity of cellular membrane, osmotic and oxidative stress, proteorhodopsin, cold-shock protein, and ice-binding protein. Additionally, in the *Flavobacterium* group with OGT ≤ 20°C, multiple amino acid residue substitutions were found, which were involved in decreasing protein stability and increasing protein flexibility. In brief, the comparison and analysis help us gain a better understanding of the microevolution and the adaptive strategies of the dominant group *Flavobacterium* in the cryosphere.

### Description of *Flavobacterium bomensis* sp. nov.

*Flavobacterium bomensis* (bo.men.′sis N.L. adj. *Bomensis* referring to Bome County, Tibetan Autonomous Region, P.R. China, from which the strains were isolated).

Cells are aerobic, Gram-negative, non-spore-forming, non-motile and non-gliding, chemoorganotrophic rods, 0.32–0.38 mm wide, and 1.04–2.08 mm long. Colonies grown on PYG agar for 9 days are circular, yellow, convex with entire margins, and about 1.5 mm in diameter. Growth occurs at 0–22°C (optimum, 10–15°C). The pH value range for growth is from 6.0 to 9.0 (optimum, pH 7.0). The salinity range for growth is from 0 to 2.5% NaCl. Catalase and oxidase are positive. No flexirubin-type pigments produced on PYG agar. Do not reduce nitrates to nitrites. The hydrolysis of gelatin and casein is variable.

No acid is produced from d-glucose, d-mannitol, inositol, l-rhamnose, d-sucrose, d-melibiose, and l-arabinose; acid production from d-sorbitol and amygdalin is variable. All strains can utilize d-glucose, d-sucrose, d-maltose, glycogen, and l-proline as sole carbon source and cannot utilize the following carbohydrate as sole carbon source: d-mannitol, l-fucose, d-sorbitol, caprate, valerate, citrate, l-histidine, 2-ketogluconate, 3-hydroxy-butyrate, 4-hydroxy-benzoate, l-rhamnose, d-ribose, inositol, itaconate, suberate, malonate, acetate, lactate, l-alanine, 5-ketogluconate, 3-hydroxy-benzoate, and l-serine. The utilization of salicin, d-melibiose, l-arabinose, N-acetyl-d-glucosamine, and propionate is variable.

Positive for starch hydrolysis, alkaline phosphatase, leucine arylamidase, valine arylamidase, and naphthol-AS-BI-phosphohydrolase. Enzyme activities of acid phosphatase and N-acetyl-*β*-glucosaminidase are variable. Negative for Voges-Proskauer test, indole and H_2_S production, esculin hydrolysis, citrate utilization, glucose fermentation, arginine dihydrolase, lysine decarboxylase, ornithine decarboxylase, urease, tryptophan deaminase, esterase (C4), esterase lipase (C8), lipase (C14), cystine arylamidase trypsin, *α*-chymotrypsin, *α*-galactosidase, *β*-galactosidase, *β*-glucuronidase, *α*-glucosidase, *β*-glucosidase, *α*-mannosidase, and *α*-fucosidase.

The most predominant cellular fatty acids are iso-C_15:0_, iso-C_15:0_ 3OH, anteiso-C_15:0_, and summed in Feature 3 (comprising C_16:1_ *ω*7*c* and/or C_16:1_ *ω*6*c*). The major polar lipid is phosphatidylethanolamine. The DNA GC content is 34.9–35.1 mol%.

Strains were isolated from melt water and ice samples on the surface of Laigu, Zepu, Renlongba, and Gawalong glaciers in Bome County, Tibetan Autonomous Region, P.R. China, in 2016. The type strain is RB1N8^T^ (= CGMCC 1.23902 = NBRC 113662).

## Author Contributions

QL and Y-HX designed the project and analyzed the data. QL, Y-HX, and Y-GZ collected the samples. QL and Y-HX purified the strains. QL performed the bioinformatic analysis of the genome sequences. H-CL performed the fatty acid analysis. QL and Y-HX wrote the manuscript.

### Conflict of Interest Statement

The authors declare that the research was conducted in the absence of any commercial or financial relationships that could be construed as a potential conflict of interest.

## Footnotes

^1^http://www.dsmz.de

^2^http://jcm.brc.riken.jp/en/

^3^http://www.cgmcc.net/
