# Supplementary material for: Microevolution and Adaptive Strategy of Psychrophilic Species Flavobacterium bomense sp. nov. Isolated From Glaciers
Source: Front Microbiol. 2019 May 22;10:1069. doi: 10.3389/fmicb.2019.01069 (PMC6538692; doi:10.3389/fmicb.2019.01069)
Supplement: Supplementary file 1 [file Data_Sheet_1.docx]

Supplementary Material

# Supplementary Figures and Tables

## Supplementary Tables

**Supplementary Table 1.** The genomes used for comparative analysis and ANI values with strain RB1N8^T^.

| **Species** | **Strain** | **Accession No.** | | **ANI values with strain RB1N8^T^** (%) |
| --- | --- | --- | --- | --- |
| *Flavobacterium bomensis* | **LB2P53** | GCA_003970305.1 | | 98.93 |
| *Flavobacterium bomensis* | **LS1R10** | GCA_003968825.1 | | 98.97 |
| *Flavobacterium bomensis* | **LS1P28** | GCA_003970335.1 | | 98.89 |
| *Flavobacterium bomensis* | **ZB4P23** | GCA_003970375.1 | | 99.02 |
| *Flavobacterium bomensis* | **RSP15** | GCA_003968755.1 | | 97.08 |
| *Flavobacterium bomensis* | **RSP46** | GCA_003984825.1 | | 98.95 |
| *Flavobacterium bomensis* | **RSP49** | GCA_003968735.1 | | 96.89 |
| *Flavobacterium bomensis* | **GSP6** | GCA_003984855.1 | | 99.08 |
| *Flavobacterium bomensis* | **GSP27** | GCA_003970395.1 | | 98.95 |
| *Flavobacterium bomensis* | **GSN2** | GCA_003984835.1 | | 98.41 |
| *Flavobacterium frigidarium* | DSM 17623^T^ | GCA_000425505.1 | | 82.59 |
| *Flavobacterium succinicans* | LMG 10402^T^ | GCA_000611675.1 | | 81.59 |
| *Flavobacterium chungangense* | LMG 26729^T^ | GCA_000735715.2 | | 81.89 |
| *Flavobacterium chilense* | LMG 26360^T^ | GCA_001602525.1 | | 81.89 |
| *Flavobacterium piscis* | CCUG 60099^T^ | GCA_001686925.1 | | 82.21 |
| *Flavobacterium crassostreae* | LPB0076^T^ | GCA_001831475.1 | | 81.97 |
| *Flavobacterium aquidurense* | DSM 18293^T^ | GCA_002217195.1 | | 82.05 |
| *Flavobacterium frigidimaris* | DSM 15937^T^ | GCA_002217275.1 | | 82.35 |
| *Flavobacterium hibernum* | ATCC 51468^T^ | GCA_002217315.1 | | 82.41 |
| *Flavobacterium hydatis* | ATCC 29551^T^ | GCA_002217335.1 | | 82.43 |
| *Flavobacterium pectinovorum* | ATCC 19366^T^ | GCA_002217365.1 | | 82.14 |
| *Flavobacterium plurextorum* | CCUG 60112^T^ | GCA_002217395.1 | | 82.18 |
| *Flavobacterium reichenbachii* | DSM 21791^T^ | GCA_002217435.1 | | 82.12 |
| *Flavobacterium spartansii* | ATCC BAA-2541^T^ | GCA_002217445.1 | | 81.88 |
| *Flavobacterium tructae* | CCUG 60100^T^ | GCA_002217475.1 | | 81.92 |
| *Flavobacterium johnsoniae* | ATCC 17061^T^ | GCA_002221975.1 | | 81.56 |
| *Flavobacterium araucananum* | DSM 24704^T^ | GCA_002222055.1 | | 82.29 |
| *Flavobacterium omnivorum* | CGMCC 1.2747^T^ | GCA_900099915.1 | | 86.29 |
| *Flavobacterium degerlachei* | DSM 15718^T^ | GCA_900106645.1 | | 83.06 |
| *Flavobacterium urumqiense* | CGMCC 1.9230^T^ | GCA_900108015.1 | | 85.56 |
| *Flavobacterium sinopsychrotolerans* | CGMCC 1.8704^T^ | GCA_900110375.1 | | 87.42 |
| *Flavobacterium frigoris* | DSM 15719^T^ | GCA_900111075.1 | | 82.97 |
| *Flavobacterium xueshanense* | CGMCC 1.9227^T^ | GCA_900112975.1 | | 87.31 |
| *Flavobacterium fluvii* | DSM 19978^T^ | GCA_900129545.1 | | 83.08 |
| *Flavobacterium micromati* | DSM 17659^T^ | GCA_900129585.1 | | 83.21 |
| *Flavobacterium granuli* | DSM 19729^T^ | GCA_900129705.1 | | 83.02 |
| *Flavobacterium saccharophilum* | DSM 1811^T^ | GCA_900142735.1 | | 82.14 |
| *Flavobacterium xinjiangense* | CGMCC 1.2749^T^ | GCA_900142885.1 | | 84.97 |
| *Flavobacterium fryxellicola* | DSM 16209^T^ | GCA_900143245.1 | | 84.8 |
| *Flavobacterium glaciei* | CGMCC 1.5380^T^ | jgi.1041405.1 | | 86.18 |
| *Flavobacterium limicola* | DSM 15094^T^ | jgi.1107685.1 | | 87.17 |
| *Flavobacterium tiangeerense* | CGMCC 1.6847^T^ | SRP106395 |  | 83.36 |

**Supplementary Table 2.** The genome information of the 11 new *Flavabacterium* strains.

|  | **Size (Mb)** | **GC (%)** | **No. of contigs** | **N50** | **L50** | **tRNA No.** | **CDS No.** | **GenBank accession No.** |
| --- | --- | --- | --- | --- | --- | --- | --- | --- |
| LB2P53 | 3.32 | 34.91 | 24 | 294740 | 4 | 42 | 3184 | RYDG00000000 |
| LS1R10 | 3.20 | 34.99 | 13 | 462765 | 2 | 41 | 3007 | RYDH00000000 |
| LS1P28 | 3.49 | 34.99 | 41 | 150318 | 7 | 41 | 3293 | RYDI00000000 |
| RB1N8 | 3.82 | 34.79 | 309 | 146079 | 8 | 43 | 3798 | RYDJ00000000 |
| ZB4P23 | 3.31 | 35.07 | 14 | 442436 | 3 | 41 | 3057 | RYDK00000000 |
| RSP15 | 3.39 | 35.09 | 30 | 224243 | 5 | 42 | 3097 | RYDL00000000 |
| RSP46 | 3.26 | 35.03 | 16 | 366060 | 3 | 41 | 3081 | RYDM00000000 |
| RSP49 | 3.43 | 35.04 | 71 | 103110 | 10 | 40 | 3138 | RYDN00000000 |
| GSP6 | 3.37 | 34.91 | 35 | 203681 | 5 | 41 | 3165 | RYDO00000000 |
| GSP27 | 3.30 | 35.02 | 30 | 201391 | 6 | 41 | 3053 | RYDP00000000 |
| GSN2 | 6.47 | 36.22 | 109 | 150760 | 13 | 76 | 6065 | RYDF00000000 |

**Supplementary Table 4.** Pan-genomes of the 10 new strains.

| **Strain** | **No. of core genes** | **No. of accessory genes** | **No. of unique genes** |
| --- | --- | --- | --- |
| LB2P53 | 2269 | 639 | 174 |
| LS1R10 | 2269 | 572 | 79 |
| LS1P28 | 2269 | 656 | 237 |
| RB1N8 | 2269 | 707 | 403 |
| ZB4P23 | 2269 | 638 | 61 |
| RSP15 | 2269 | 566 | 172 |
| RSP46 | 2269 | 577 | 125 |
| RSP49 | 2269 | 537 | 221 |
| GSP6 | 2269 | 683 | 110 |
| GSP27 | 2269 | 608 | 75 |

**Supplementary Table 4.** Differences of carbon source utilization between the 11 new strains tested by API ID 32 GN.

Strains: 1, LB2P53; 2, LS1R10; 3, LS1P28; 4, RB1N8^T^; 5, ZB4P23; 6, RSP15; 7, RSP46; 8, RSP49; 9, GSP6; 10, GSP27; 11, GSN2. +, Positive; –, negative.

| **Utilization of:** | **1** | **2** | **3** | **4** | **5** | **6** | **7** | **8** | **7** | **10** | **11** |
| --- | --- | --- | --- | --- | --- | --- | --- | --- | --- | --- | --- |
| D-glucose | + | + | + | + | + | + | + | + | + | + | + |
| salicin | - | + | + | + | + | w | + | + | w | + | + |
| D-melibiose | - | - | - | - | - | + | + | + | w | w | - |
| L-arabinose | - | - | - | - | - | - | - | + | - | - | - |
| propionate | - | - | - | - | - | - | w | - | - | - | - |
| L-proline | + | + | + | + | + | + | + | + | + | + | + |
| N-acetyl-D-glucosamine | - | - | - | - | - | + | - | + | + | + | + |
| D-sucrose | + | + | + | + | + | + | + | + | + | + | + |
| D-maltose | + | + | + | + | + | + | + | + | + | + | + |
| glycogen | + | + | + | + | + | + | + | + | + | + | + |

**Supplementary Table 5.** Genes with known or predicted roles in cold adaptation and growth present in genomes of the new psychrophilic species.

Strains: 1, LB2P53; 2, LS1R10; 3, LS1P28; 4, RB1N8^T^; 5, ZB4P23; 6, RSP15; 7, RSP46; 8, RSP49; 9, GSP6; 10, GSP27; 11, *F. granuli* DSM 19729^T^; 12, *F. crassostreae* LPB0076^T^.

| **Product name** | **Gene symbol** | COGs | **1** | **2** | **3** | **4** | **5** | **6** | **7** | **8** | **7** | **10** | **11** | **12** |
| --- | --- | --- | --- | --- | --- | --- | --- | --- | --- | --- | --- | --- | --- | --- |
| **Proteorhodopsin** |  |  |  |  |  |  |  |  |  |  |  |  |  |  |
| Proteorhodopsin | *PR* |  | 1 | 1 | 1 | 1 | 1 | 1 | 1 | 1 | 1 | 1 | 0 | 0 |
| Predictet Brp-like protein Blh | *Blh* |  | 1 | 1 | 1 | 1 | 1 | 1 | 1 | 1 | 1 | 1 | 0 | 0 |
| **Transporter** |  |  |  |  |  |  |  |  |  |  |  |  |  |  |
| Lipopolysaccharide export system protein LptA | *lptA* |  | 1 | 1 | 1 | 1 | 1 | 1 | 1 | 1 | 1 | 1 | 0 | 0 |
| Lipopolysaccharide ABC transporter, ATP-binding protein LptB | *lptB* |  | 1 | 1 | 1 | 1 | 1 | 1 | 1 | 1 | 1 | 1 | 0 | 0 |
| Lipopolysaccharide export system protein LptC | *lptC* |  | 1 | 1 | 1 | 1 | 1 | 1 | 1 | 1 | 1 | 1 | 0 | 0 |
| Lipopolysaccharide export system permease protein LptF | *lptF* |  | 1 | 1 | 1 | 1 | 1 | 1 | 1 | 1 | 1 | 1 | 0 | 0 |
| Lipopolysaccharide export system permease protein LptG | *lptG* |  | 1 | 1 | 1 | 1 | 1 | 1 | 1 | 1 | 1 | 1 | 0 | 0 |
| **Ice-binding protein** |  |  | 1 | 1 | 1 | 1 | 1 | 1 | 1 | 1 | 1 | 1 | 0 | 0 |
| **SIGMA FACTORS** |  |  |  |  |  |  |  |  |  |  |  |  |  |  |
| DNA-directed RNA polymerase sigma 24 | *rpoE* | COG1595 | 9 | 10 | 10 | 11 | 9 | 10 | 9 | 9 | 9 | 9 | 9 | 4 |
| DNA-directed RNA polymerase, sigma 70 | *rpoD* | COG0568 | 1 | 1 | 1 | 1 | 1 | 1 | 1 | 1 | 1 | 1 | 1 | 1 |
| **OXIDATIVE STRESS RESPONSE** | |  |  |  |  |  |  |  |  |  |  |  |  |  |
| Superoxide dismutase | *sodA* | COG0605 | 4 | 3 | 4 | 5 | 4 | 4 | 3 | 4 | 4 | 4 | 2 | 2 |
| Catalase | *katE* | COG0753 | 1 | 1 | 1 | 1 | 1 | 1 | 1 | 1 | 1 | 1 | 1 | 0 |
| Catalase (peroxidase I) | *katG* | COG0376 | 1 | 1 | 1 | 3 | 1 | 1 | 1 | 1 | 1 | 1 | 0 | 0 |
| Peroxiredoxin | *bcp* | COG1225 | 3 | 3 | 3 | 3 | 3 | 3 | 3 | 3 | 3 | 3 | 1 | 1 |
| Organic hydroperoxide reductase | *osmC/ohr* | COG1764 | 3 | 2 | 2 | 3 | 2 | 2 | 2 | 2 | 2 | 2 | 0 | 0 |
| Thioredoxin reductase | *trxB* | COG0492 | 3 | 3 | 3 | 4 | 3 | 3 | 3 | 4 | 3 | 3 | 3 | 2 |
| Thiol-disulfide isomerase and thioredoxins | *trxA* | COG0526 | 9 | 8 | 9 | 8 | 11 | 9 | 9 | 9 | 9 | 10 | 4 | 8 |
| **COLD SHOCK RESPONSE** |  |  |  |  |  |  |  |  |  |  |  |  |  |  |
| Cold shock protein | *cspA* | COG1278 | 1 | 1 | 1 | 1 | 1 | 1 | 1 | 1 | 1 | 1 | 1 | 1 |
| Transcription elongation factor | *nusA* | COG0195 | 1 | 1 | 1 | 1 | 1 | 1 | 1 | 1 | 1 | 1 | 1 | 1 |
| Polyribonucleotide nucleotidyltransferase | *pnp* | COG1185 | 1 | 1 | 1 | 1 | 1 | 1 | 1 | 1 | 1 | 1 | 1 | 1 |
| Ribosome-binding factor A | *rbfA* | COG0858 | 1 | 1 | 1 | 1 | 1 | 1 | 1 | 1 | 1 | 1 | 1 | 1 |
| Translation initiation factor 1 | *infA* | COG0361 | 1 | 1 | 1 | 1 | 1 | 1 | 1 | 1 | 1 | 1 | 1 | 1 |
| Translation initiation factor 2 | *infB* | COG0532 | 1 | 1 | 1 | 1 | 1 | 1 | 1 | 1 | 1 | 1 | 1 | 1 |
| **MEMBRANE ADAPTATIONS** |  |  |  |  |  |  |  |  |  |  |  |  |  |  |
| Linoleoyl-CoA desaturase |  | COG3239 | 1 | 1 | 1 | 1 | 1 | 1 | 1 | 1 | 1 | 1 | 1 | 1 |
| acyl-ACP desaturase | *desA2* |  | 1 | 1 | 1 | 1 | 1 | 1 | 1 | 1 | 1 | 1 | 1 | 0 |
| **Carotenoid biosynthesis** |  |  |  |  |  |  |  |  |  |  |  |  |  |  |
| Isopentenyl-diphosphate Delta-isomerase | *idi* | COG1443 | 1 | 1 | 1 | 1 | 1 | 1 | 1 | 1 | 1 | 1 | 1 | 1 |
| Beta-carotene hydroxylase | *crtZ* |  | 1 | 1 | 1 | 1 | 1 | 1 | 1 | 1 | 1 | 1 | 1 | 1 |
| Phytoene synthase | *crtB* | COG1562 | 1 | 1 | 1 | 1 | 1 | 1 | 1 | 1 | 1 | 1 | 1 | 1 |
| Phytoene dehydrogenase | *crtI* | COG1233 | 1 | 1 | 1 | 1 | 1 | 1 | 1 | 1 | 1 | 1 | 1 | 1 |
| Lycopene beta cyclase | *crtY* |  | 1 | 1 | 1 | 1 | 1 | 1 | 1 | 1 | 1 | 1 | 1 | 1 |
| **OSMOPROTECTION** |  |  |  |  |  |  |  |  |  |  |  |  |  |  |
| **Glycogen metabolism** |  |  |  |  |  |  |  |  |  |  |  |  |  |  |
| Glycogen synthase | *glgA* | COG0297 | 2 | 2 | 2 | 2 | 2 | 2 | 2 | 2 | 2 | 2 | 2 | 2 |
| 1,4-alpha-glucan branching enzyme | *glgB* | COG0296 | 2 | 2 | 2 | 2 | 2 | 2 | 2 | 2 | 2 | 2 | 2 | 1 |
| Glucose-1-phosphate adenylyltransferase | *glgC* | COG0448 | 1 | 1 | 2 | 1 | 2 | 2 | 1 | 2 | 2 | 2 | 1 | 1 |
| **Proline synthesis** |  |  |  |  |  |  |  |  |  |  |  |  |  |  |
| Gamma-glutamyl phosphate reductase | *proA* | COG0014 | 1 | 1 | 1 | 1 | 1 | 1 | 1 | 1 | 1 | 1 | 1 | 0 |
| Glutamate 5-kinase | *proB* | COG0263 | 1 | 1 | 1 | 1 | 1 | 1 | 1 | 1 | 1 | 1 | 1 | 0 |
| Pyrroline-5-carboxylate reductase | *proC* | COG0345 | 1 | 1 | 1 | 1 | 1 | 1 | 1 | 1 | 1 | 1 | 1 | 1 |
| Na+/proline symporter | *putP* | COG0591 | 4 | 4 | 4 | 4 | 5 | 5 | 5 | 4 | 4 | 5 | 2 | 1 |
| **Others** |  |  |  |  |  |  |  |  |  |  |  |  |  |  |
| tRNA-dihydrouridine synthase | *dusB* | COG0042 | 2 | 2 | 2 | 2 | 2 | 2 | 2 | 2 | 2 | 2 | 2 | 2 |
| **CRISPR system** |  |  |  |  |  |  |  |  |  |  |  |  |  |  |
| CRISPR-associated protein Cas1 | *cas1* |  | 1 | 1 | 1 | 1 | 1 | 1 | 1 | 1 | 1 | 1 | 1 | 1 |
| CRISPR-associated protein Cas2 | *cas2* |  | 1 | 1 | 1 | 1 | 1 | 1 | 1 | 1 | 1 | 1 | 1 | 1 |
| CRISPR-associated endonuclease Cas9 | *cas9* |  | 1 | 1 | 1 | 1 | 1 | 1 | 1 | 1 | 1 | 1 | 2 | 0 |
| CRISPR repeat (35-36bp) |  |  | 45 | 44 | 15 | 52 | 9 | 31 | 45 | 34 | 26 | 62 |  |  |

**Supplementary Table 6.** Cellular fatty acids compositions (%) of the 11 new strains and related strains.

Strains: 1, RB1N8^T^; 2, LB2P53; 3, LS1R10; 4, LS1P28; 5, ZB4P23; 6,RSP15; 7, RSP46; 8, RSP49; 9, GSP6; 10, GSP27; 11, GSN2; 12, *F*. *urumqiense* CGMCC 1.9230^T^; 13, *F*. *sinopsychrotolerans* CGMCC 1.8704^T^; 14, *F*. *tiangeerense* CGMCC 1.6847^T^; 15, *F*. *xueshanense* CGMCC 1.9227^T^; 16, *F*. omnivorum CGMCC 1.2747^T^; 17, *F*. *frigidarium* CGMCC 1.9172^T^. Values are percentages of the total fatty acids. Tr, traces (less than 1% of the total fatty acids); -, not detected.

| **Fatty acids** | **1** | **2** | **3** | **4** | **5** | **6** | **7** | **8** | **9** | **10** | **11** | **12** | **13** | **14** | **15** | **16** | **17** |
| --- | --- | --- | --- | --- | --- | --- | --- | --- | --- | --- | --- | --- | --- | --- | --- | --- | --- |
| iso-C_13:0_ | Tr | Tr | Tr | Tr | Tr | Tr | Tr | Tr | Tr | Tr | Tr | 1.15 | - | 1.11 | - | - | Tr |
| C_14:0_ | 1.2 | 1.2 | 1.0 | 1.1 | Tr | Tr | 1.2 | Tr | Tr | Tr | 1.0 | Tr | Tr | Tr | Tr | Tr | - |
| iso-C_14:0_ | 2.3 | 2.6 | 2.8 | 2.0 | 3.2 | 2.0 | 1.9 | 2.4 | 3.0 | 2.8 | 2.8 | 6.1 | 3.2 | 3.2 | 4.2 | 2.8 | 1.9 |
| iso-C_14:0_ 3OH | Tr | Tr | Tr | Tr | Tr | Tr | Tr | Tr | Tr | Tr | Tr | Tr | Tr | Tr | 1.1 | Tr | Tr |
| C_15:0_ 3OH | - | - | - | - | 1.1 | - | 1.1 | - | - | - | - | 1.1 | - | - | 1.4 | - | - |
| **iso-C_15:0_** | **16.4** | **13.9** | **17.3** | **16.9** | **17.4** | **14.7** | **15.2** | **15.2** | **15.5** | **17.1** | **16.0** | **7.7** | **10.0** | **16.3** | **5.3** | **11.8** | **17.4** |
| iso-C_15:1_ G | 3.7 | 3.2 | 3.5 | 4.7 | 4.5 | 3.2 | 3.0 | 6.4 | 4.9 | 3.5 | 4.2 | 3.1 | 5.3 | 5.6 | 3.6 | 5.8 | 7.6 |
| iso-C_15:0_ 3OH | 6.9 | 7.2 | 6.7 | 7.2 | 7.6 | 5.9 | 8.3 | 7.5 | 7.3 | 7.4 | 7.1 | 3.1 | 7.6 | 11.3 | 3.3 | 6.5 | 9.7 |
| anteiso-C_15:0_ | 6.3 | 8.5 | 7.4 | 6.9 | 5.4 | 11.6 | 4.8 | 7.6 | 8.0 | 8.4 | 6.0 | 4.8 | 10.2 | 4.9 | 1.3 | 6.4 | 5.0 |
| anteiso-C_15:1_ A | Tr | Tr | Tr | Tr | Tr | Tr | Tr | Tr | Tr | Tr | Tr | Tr | 1.3 | Tr | Tr | Tr | Tr |
| C_15:1_ *ω*6*c* | 3.0 | 2.8 | 2.7 | 2.0 | 3.8 | 2.8 | 2.4 | 4.7 | 3.0 | 3.0 | 3.6 | 11.4 | 5.7 | 4.8 | 8.5 | 3.5 | 4.3 |
| C_16:0_ | 4.0 | 2.4 | 3.2 | 4.4 | 2.7 | 1.6 | 3.0 | 1.6 | 2.8 | 2.5 | 3.0 | 1.2 | Tr | Tr | 1.2 | 2.7 | 3.0 |
| C_16:0_ 3OH | 3.3 | 2.8 | 2.5 | 2.7 | 2.6 | Tr | 3.7 | ND | 2.2 | 2.0 | 2.7 | 1.2 | Tr | Tr | 1.5 | 1.5 | 2.3 |
| iso-C_16:0_ | 3.2 | 2.4 | 3.4 | 3.2 | 3.3 | 3.7 | 1.8 | 2.7 | 4.0 | 2.9 | 3.0 | 7.5 | 3.4 | 3.3 | 11.9 | 6.2 | 3.2 |
| iso-C_16:1_ H | 2.4 | 2.9 | 2.4 | 2.2 | 2.3 | 4.7 | 1.3 | 3.8 | 2.8 | 2.6 | 2.6 | 8.2 | 3.6 | 4.9 | 12.6 | 4.4 | 1.9 |
| iso-C_16:0_ 3OH | 4.1 | 5.1 | 4.7 | 3.7 | 5.5 | 6.2 | 3.9 | 5.8 | 6.1 | 4.6 | 5.0 | 11.0 | 11.8 | 9.2 | 15.9 | 8.7 | 3.4 |
| iso-C_17:0_ 3OH | 4.6 | 4.4 | 5.3 | 5.6 | 4.6 | 7.6 | 4.5 | 5.4 | 5.1 | 5.1 | 4.5 | 2.2 | 6.2 | 4.8 | 3.3 | 6.0 | 6.9 |
| C_17:1_ *ω*6*c* | 2.3 | 2.6 | 2.3 | 1.2 | 3.2 | 2.2 | 2.0 | 3.8 | 2.3 | 2.6 | 3.1 | 6.1 | 9.4 | 7.0 | 9.2 | 4.6 | 2.3 |
| C_18:1_ *ω*5*c* | Tr | Tr | Tr | 1.0 | Tr | 1.1 | Tr | Tr | Tr | 1.2 | Tr | Tr | 1.4 | 1.2 | Tr | Tr | Tr |
| Summed feature 2 | Tr | Tr | Tr | Tr | Tr | - | 1.1 | - | Tr | Tr | Tr | - | - | Tr | - | - | - |
| **Summed feature 3** | **28.3** | **29.1** | **25.2** | **26.6** | **24.1** | **17.4** | **33.8** | **20.6** | **23.3** | **24.3** | **26.3** | **17.9** | **9.4** | **11.2** | **11.0** | **17.5** | **23.1** |
| Summed feature 4 | 1.1 | 1.0 | Tr | Tr | - | 1.6 | - | 1.9 | 1.2 | 1.2 | 1.2 | - | 1.9 | 1.7 | - | 1.3 | 1.4 |
| Summed feature 9 | 2.5 | 2.4 | 3.1 | 3.9 | 2.4 | 7.5 | 2.4 | 5.2 | 2.9 | 3.4 | 2.9 | 1.2 | 3.0 | 3.1 | 1.5 | 4.5 | 2.7 |

*Summed features represent groups of two or three fatty acids that could not be separated by gas chromatography with the MIDI system. Summed in feature 2 contains C_14:0_ 3OH and/or iso-C_16:1_ I; Summed in feature 3 contains C_16:1_*ω*6c and/or C_16:1_*ω*7*c*; Summed in feature 4 contains iso-C_17:1_ I/anteiso-C_17:1_ B; Summed in feature 9 contains iso-C_17:1_*ω*9*c* or 10-methyl C_16:0_.

**Supplementary Table 7.** Differential characteristics between *Flavabacterium bomense* sp. nov. and the type strains of related species of the genus *Flavobacterium*.

Strains: 1, *F. bomense* sp. nov. (11 strains); 2, *F. urumqiense* CGMCC 1.9230^T^; 3, *F. sinopsychrotolerans* CGMCC 1.8704^T^; 4, *F. tiangeerense* CGMCC 1.6847^T^; 5, *F.* *xueshanense* CGMCC 1.9227^T^; 6, *F. omnivorum* CGMCC 1.2747^T^；7，*F. frigidarium* CGMCC 1.9172^T^. +, Positive; –, negative.

| **Characteristics** | **1** | **2** | **3** | **4** | **5** | **6** | **7** |
| --- | --- | --- | --- | --- | --- | --- | --- |
| Maximum growth temperature (°C) | 17-22 | 17 | 25 | 26 | 18 | 20 | 24 |
| Maximum growth with NaCl (%) | 2.0-2.5 | 0.5 | 2.0 | 0.5 | 0.5 | 3.5 | 5 |
| arginine dihydrolase | - | - | + | - | - | - | - |
| citrate utilization | - | - | + | - | - | - | - |
| **Hydrolysis of:** |  |  |  |  |  |  |  |
| ESC aesculin | - | + | + | + | + | + | + |
| Starch | + | -^#^ | +^#^ | -^#^ | -^#^ | +^#^ | -^#^ |
| **Utilization of:** |  |  |  |  |  |  |  |
| D-mannitol | - | - | - | - | - | - | + |
| D-glucose | + | + | + | + | - | + | + |
| L-proline | + | - | + | - | - | + | + |
| D-sucrose | + | + | + | + | + | + | - |
| glycogen | + | - | + | - | - | + | - |
| L-serine | - | - | + | - | - | - | - |
| **Enzyme activities (API ZYM):** |  |  |  |  |  |  |  |
| cystine arylamidase | - | - | + | - | - | - | - |
| α-chymotrypsin | - | - | + | - | - | - | - |
| α-glucosidase | - | + | + | + | - | + | - |

## Supplementary Figures


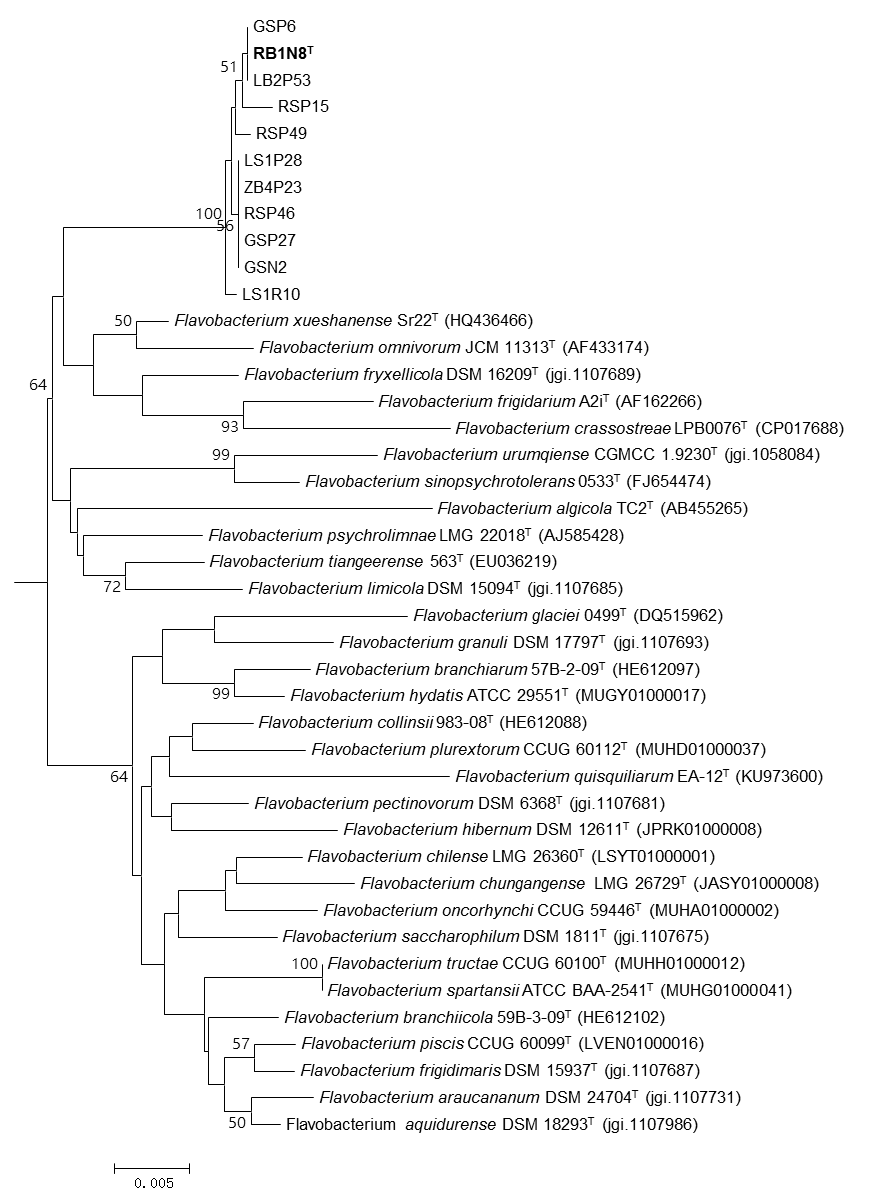


**Supplementary Figure 1.** Neighbor-joining tree of strain RB1N8^T^ and related taxa based on 16S rRNA gene sequence comparisons. Bootstrap values (≥50%) based on 1000 replicates are shown at branch points. Bar, 0.005 nt substitutions per site.


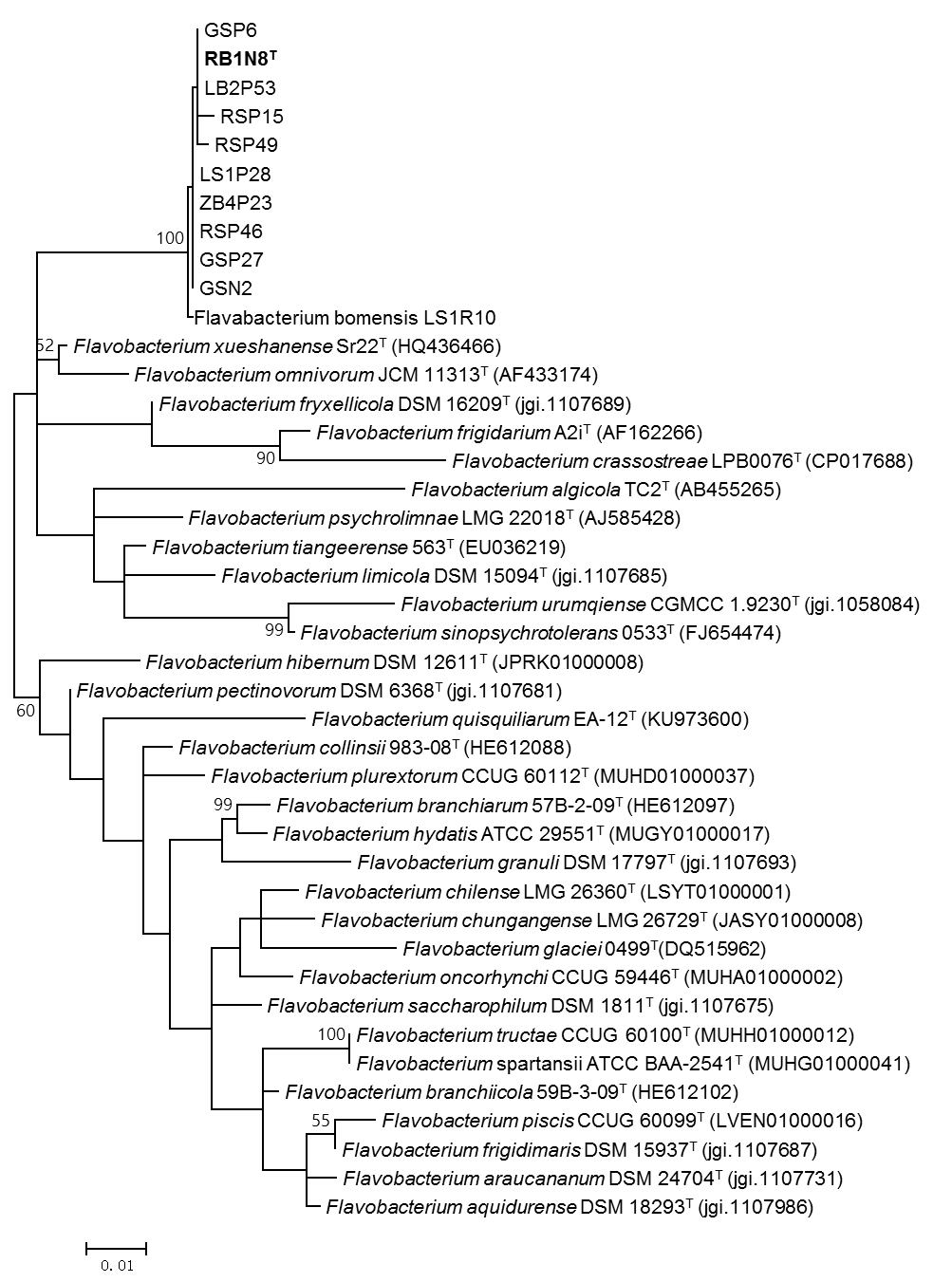
**Supplementary Figure 2.** Maximum-likelihood tree of strain RB1N8^T^ and related taxa based on 16S rRNA gene sequence comparisons. Bootstrap values (≥50%) based on 1000 replicates are shown at branch points. Bar, 0.01 nt substitutions per site.


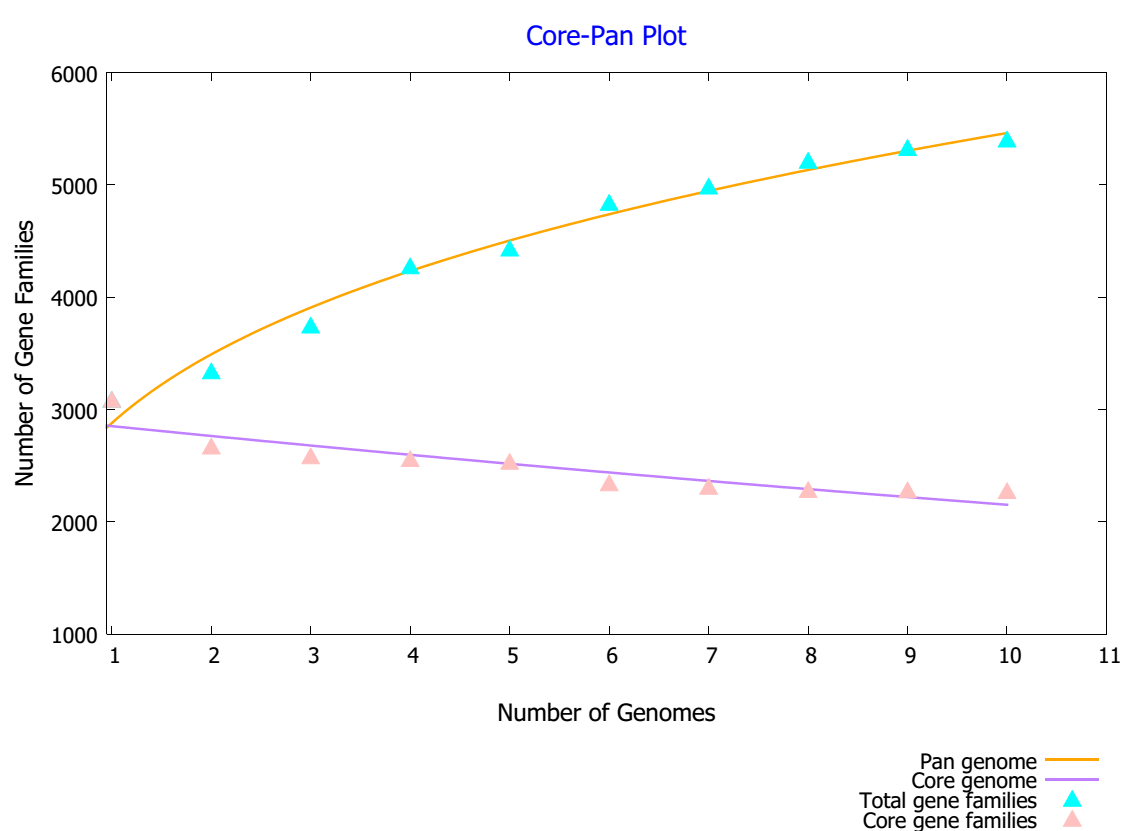


**Supplementary Figure 3.**  Pan-genome analysis of *Flavabacterium bomense* sp. nov., showing the pan-genome curve generated by plotting the total number of distinct gene families in the pan-genome and the core genome against the number of genomes considered. The analysis was performed using the BPGA tool with default parameters.

**Supplementary Figure 4.**
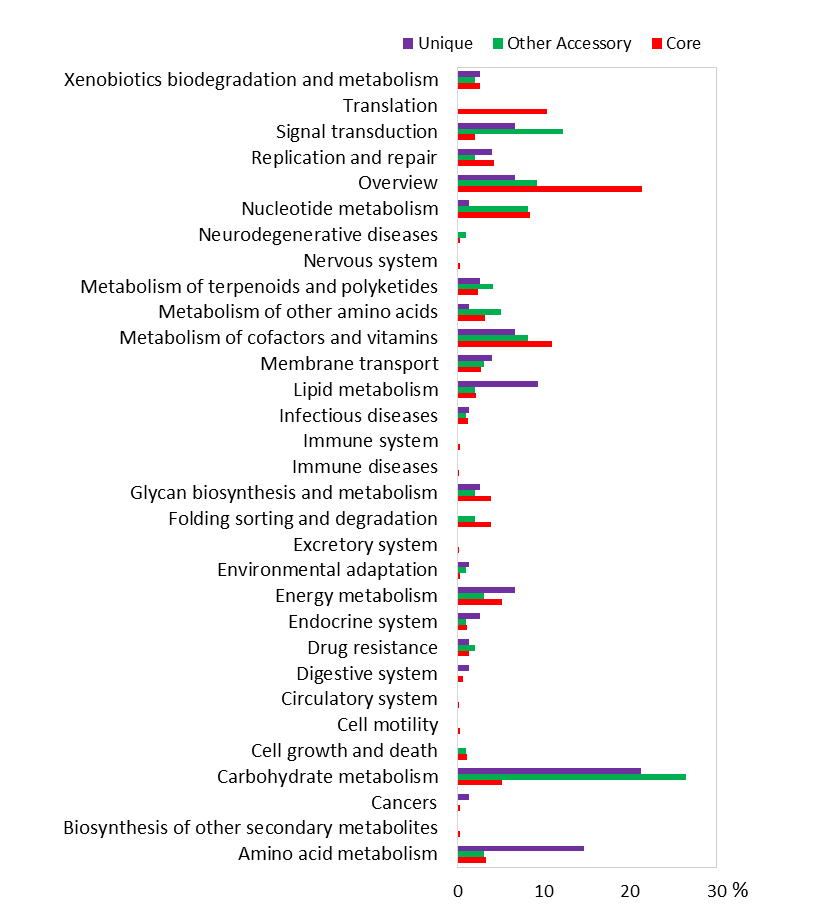
 KEGG distribution for the pan genome of 10 glacier-inhabiting *Flavabacterium* strains determined using the BPGA tool with default parameters. The x axis represents the relative percentage of the number of genes in each KEGG category.


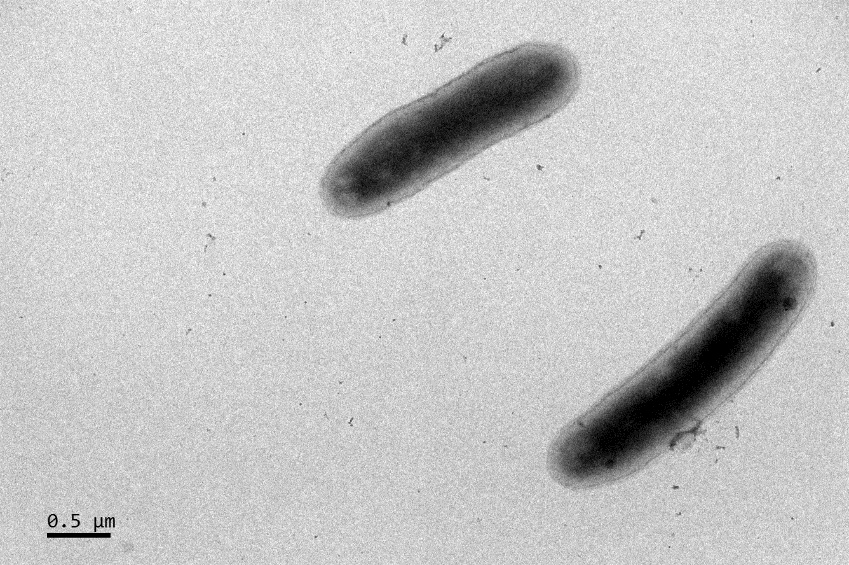


**Supplementary Figure 5.** Transmission electron micrograph of strain RB1N8^T^ grown on PYG agar for 3 days at 14°C. Bar, 0.5 μm.


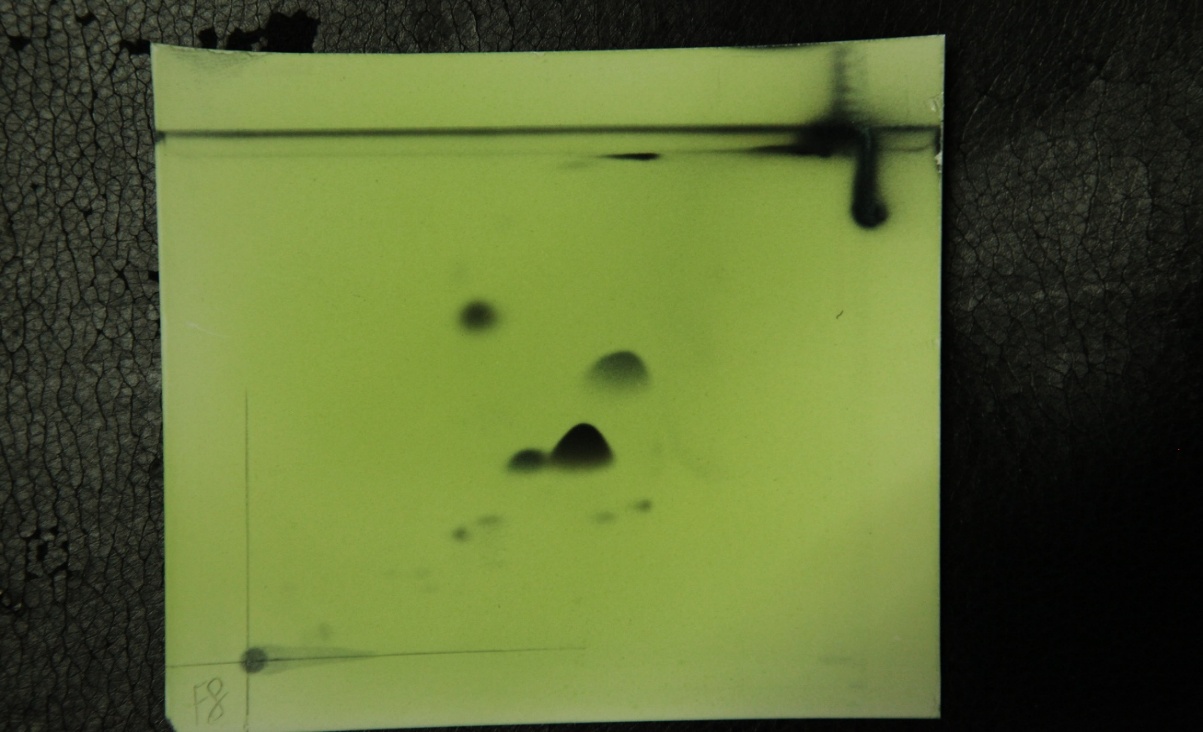


**UL**

**UL**

**PE**

**AL**

**AL**

**AL**

**AL**

**UL**

**Supplementary Figure 6.** Total polar lipid distribution of strain RB1N8^T^ after spraying with 5% ethanolic olybdatophosphoric acid.

PE, phosphatidylethanolamine; AL, unidentified aminolipid; UL, unidentified lipid.
